# Does communication help people coordinate?

**DOI:** 10.1371/journal.pone.0170780

**Published:** 2017-02-08

**Authors:** Yevgeniy Vorobeychik, Zlatko Joveski, Sixie Yu

**Affiliations:** Electrical Engineering and Computer Science, Vanderbilt University, Nashville, TN, United States of America; Middlesex University, UNITED KINGDOM

## Abstract

Theoretical and experimental investigations have consistently demonstrated that collective performance in a variety of tasks can be significantly improved by allowing communication. We present the results of the first experiment systematically investigating the value of communication in networked consensus. The goal of all tasks in our experiments is for subjects to reach global consensus, even though nodes can only observe choices of their immediate neighbors. Unlike previous networked consensus tasks, our experiments allow subjects to communicate either with their immediate neighbors (locally) or with the entire network (globally). Moreover, we consider treatments in which essentially arbitrary messages can be sent, as well as those in which only one type of message is allowed, informing others about a node’s local state. We find that local communication adds minimal value: fraction of games solved is essentially identical to treatments with no communication. Ability to communicate globally, in contrast, offers a significant performance improvement. In addition, we find that constraining people to only exchange messages about local state is significantly better than unconstrained communication. We observe that individual behavior is qualitatively consistent across settings: people clearly react to messages they receive in all communication settings. However, we find that messages received in local communication treatments are relatively uninformative, whereas global communication offers substantial information advantage. Exploring mixed communication settings, in which only a subset of agents are global communicators, we find that a significant number of global communicators is needed for performance to approach success when everyone communicates globally. However, global communicators have a significant advantage: a small tightly connected minority of globally communicating nodes can successfully steer outcomes towards their preferences, although this can be significantly mitigated when all other nodes have the ability to communicate locally with their neighbors.

## Introduction

Coordination is central to many biological processes, such as drafting in flocks of birds and schools of fish, huddling in groups of penguins [1], and groups of ants working together to move a large object [2] or building bridges with their own bodies allowing others to cross a gap in their foraging trail [3]. Coordination is also an intrinsic part of many human tasks, such as successful navigation in large crowds and coordination of cockpit members to ensure flight safety [4]. Consequently, there is considerable literature that explores mechanisms that facilitate better coordination [4, 5].

It is widely believed that communication is one of the key mechanisms promoting coordination among people [6–11]. Some, indeed, view it as a crucial driver behind the evolution of language [6, 7]. For example, Szamado [6] argues that the complexity of recruitment and coordination of group hunting provided an important impetus for development of early language. The experiment of Selten et al. [11] demonstrates that a simple symbolic language can indeed emerge in the context of a coordination task in which a common language is explicitly ruled out at the beginning. More broadly, there have been a number of theoretical and experimental studies of how communication contributes to the effectiveness of a variety of coordination tasks. On the theoretical front, most efforts consider the impact of communication on selected equilibria in two-player coordination games [12–17]. For example, Farrell [12] shows that a simple model of pre-play communication that is costless, nonbinding, and nonverifiable (cheap talk), results in greater coordination in a battle-of-the-sexes game. More recently, Demichelis et al. [15] show that by associating messages with actions taken in the coordination game, and positive preferences for honesty, evolutionary stable outcomes lead to efficient coordination. Ellingsen et al. [16] use a level-k reasoning model, built around the presumption that subjects’ strategic behavior can be classified into different levels of reasoning based on their beliefs about opponents’ behavior, to offer a general characterization of the value of communication in symmetric 2x2 games, showing that it is helpful in common-interest games with positive spillovers and strategic complementarities. Experimental literature on the value of communication in coordination has followed most theoretical models, separating the communication phase, in which all players get to talk to each other, followed by the actual coordination task, typically involving two players playing a game such as the battle-of-the-sexes or stag hunt. Cooper et al. [18] evaluate effectiveness of one-way (single talker) and two-way (both players communicating with each other) communication preceding two-player games. In their experiments, messages were restricted to action intentions, and they found that communication typically increased frequency of successful coordination. The review of social dilemma research by Dawes [19] describes successful use of communication to promote coordination in social dilemma games. Recently, Choi et al. [20] considered the impact of networks restricting pre-play communication on success in the subsequent (not networked) coordination task. These studies complement a significantly larger theoretical and experimental literature on human coordination, including work by Kearns et al. [21–23], as well as a number of related efforts characterizing diffusion of ideas, conformity, and preferences on networks [24–27].

In most of the prior literature, theoretical or experimental, communication has been grafted on as a distinct pre-play stage. Moreover, experimental focus has been on simple, two-player games. The prognosis has been overwhelmingly positive: communication has been shown to promote better coordination, across different tasks. However, both the segregation of communication into a distinct phase, and the dominant focus on games with only two players, are quite simplistic. Many real coordination tasks involve a significantly larger number of parties (for example, successfully hunting big game may require groups of at least 5 [6]), and, critically, coordination and communication are interleaved within a task. Moreover, coordination on social networks may restrict the ability to communicate to be among network neighbors; indeed, this is typical of social media settings.

We investigate the role of communication in a far more complex networked coordination task, involving 20 players situated as nodes on a network who can make decisions in real-time over a fixed time horizon, but only observe decisions by their network neighbors (nearly instantly after these are made). We build on a class of such games introduced by Kearns et al. [21–23], considering global consensus as the goal, but allowing strategic tension akin to battle-of-the-sexes. Unlike most prior experimental literature, we embed communication directly into the task itself, allowing subjects to communicate as well as make decisions in real time. Moreover, we consider the impact of (a) communication restricted to local neighborhoods, or (b) global communication, as well as the role of strong constraints on the information content of messages, on the ability of people to ultimately reach global consensus, with or without strategic tension. Perhaps our most surprising finding is that communication *need not improve coordination*: in particular, local communication does not lead to greater likelihood of coordination success. Global communication, in contrast, significantly improves the ability of people to coordinate. Interestingly, this finding is consistent whether or not there exists a strategic tension. We consider two hypotheses to explain this: first, that human behavior is qualitatively different across the communication treatments, and second, that local communication does not entail sufficient information content to improve coordination success. By analyzing both individual (micro-) behavior, and marginal information of messages received, we find that the second hypothesis receives the most support: for example, humans appear to respond to messages in qualitatively similar ways across treatments, whereas global communication leads to significantly more informative messages. In a series of further investigations we additionally explore the impact of mixed communication (where some players communicate locally and other globally) and considerations of equity in outcomes when only a minority of players can communicate globally and can steer outcomes towards their preferences in opposition of the majority.

## Experimental methodology

**Fig 1:**
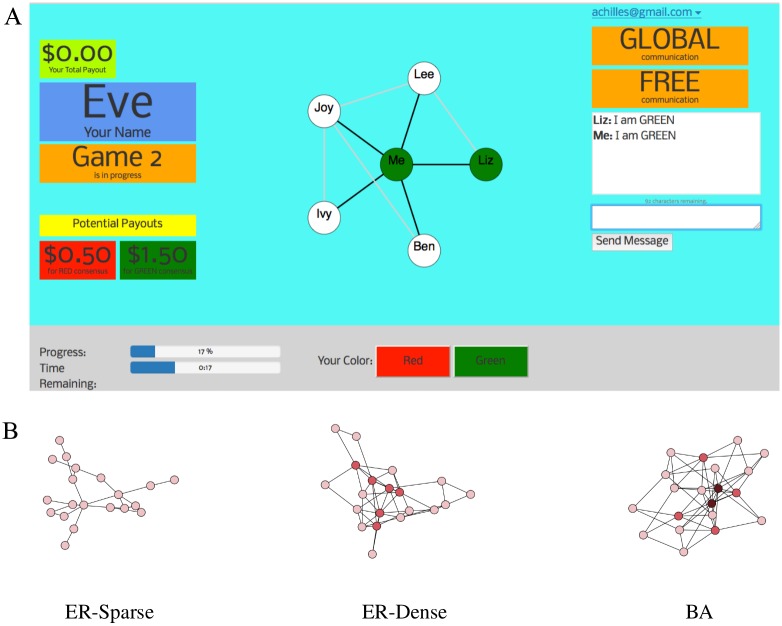
**A**: an example graphical interface from the point of view of an experimental subject, who is represented by a node in the network. The subject can see both her own node (labeled as “Me”) and her network neighbors (labeled with their pseudonyms, randomly assigned at the beginning of a game), as well as connections among her neighbors. The subject can also observe her current total payout in the experiment session (over all games played thus far). In some treatments individuals had preferences about which color is chosen in consensus; these preferences are shown on the left. Also on the bottom left portion of the interface the subjects see both progress towards global consensus, as well as time remaining in the game. Finally, in games involving communication, an instant-message-like interface is shown on the right, with a box where messages can be viewed and entered. A clearly labeled sign describes whether the game involves LOCAL or GLOBAL communication. **B**: example instances of networks, where darker colors indicate higher node degrees.

We designed experiments in which sets of 20 human subjects, situated as nodes on a network, chose one of two colors (red or green), with the primary goal of reaching global consensus on a single color, echoing several prior experiments in networked consensus [21–23]. In all experiments, individuals (acting as nodes) could see color choices by their network neighbors (or white, if no choice had been made), and could change their own color at any point. The crucial element of these experiments which is entirely novel is the integration of an instant-message-like communication interface (see Fig 1).

First we recruited workers from Amazon’s Mechanical Turk, a crowdsourcing site. In the recruitment stage, each worker completed a simple three-question English language proficiency test, followed by a tutorial that explained each part of the experimental setup, the networked consensus task, and the application interface. Once they completed the tutorial, participants were required to affirm (by checking a box in an online form) that they were over the age of 18 and that they had read and consented to the terms of participation. We also asked participants to indicate their time availability and if they would agree to be contacted for scheduled experiment sessions. Workers were able to participate in a full experiment session only after they have completed the recruitment stage. The Vanderbilt University Institutional Review Board reviewed this informed consent procedure and approved it, along with the overall experimental protocol.

Altogether, we ran 239 such experiments over 6 sessions, involving 131 distinct participants. Each game lasted at most 60 seconds, and terminated as soon as consensus was reached. Each participant received $0.15 for each game. In addition, if the game reached consensus each player received a bonus. The magnitude of the bonus depended on two things: 1) whether the game involved individuals with color preferences, and 2) which color was chosen as consensus. In the first case, no matter which color was chosen in consensus, all subjects received $0.20. In the second case, if consensus was achieved in which all players chose the individual’s preferred color, this participant received a $0.30 bonus; if, on the other hand, the less preferred color was chosen in consensus, the bonus to this individual was only $0.10. In all treatments involving color preferences (which constituted half of all treatments), exactly 10 nodes preferred each of the two colors.

In our experiments we systematically varied four factors: a) communication form, b) communication structure, c) network structure, and d) color preferences. Communication form involved three treatments: no communication, which provided our baseline, local communication, where individuals could only exchange messages within their immediate neighborhood, and global communication, which allowed messages to be seen by the entire network. In communication structure, on the other hand, we manipulated the extent to which actual messages sent were constrained through two treatments: unconstrained, in which arbitrary natural language (or otherwise) messages could be exchanged, and constrained, in which only messages of the form “# neighbors choosing green, # neighbors choosing red” could be sent. The “unconstrained” treatments did involve two character-limit constraints: we imposed a 10-character limit on each message, and a 50-character limit on all messages sent by a given node in a game. In the experiments, these character limits appeared to be quite generous.

We varied network structure among three categories: sparse and dense Erdos-Renyi networks (Sparse- and Dense-ER, respectively), in which connectivity is entirely random, and Barabasi-Albert preferential attachment networks (BA), where connectivity is heavily skewed towards high-degree nodes. Finally, we considered settings with no color preferences, and those in which different participants faced conflicting preferences about colors (for example, some receiving a higher payout for a red, and others for a green, consensus); however, the number of subjects preferring each color was always equal.

Finally, to ensure quality of the data, we treated games in which one or more participants did not choose a color at all as invalid, and removed these from consideration. In the end, we were left with 239 valid games which comprised our first analysis.

Prior research considering the role of communication in coordination has almost universally found that allowing people to communicate improves their performance. However, most such investigations were either not tightly controlled, were very small-scale, or embedded communication as a distinct phase, in which all individuals were allowed to discuss the task. Our setup aims to more accurately reflect realistic role that communication plays in coordination by embedding it directly within the task itself, and varying it across two dimensions: form (local vs. global) and structure (unconstrained vs. constrained).

## Results

### The value of communication: Local vs. global

**Fig 2:**
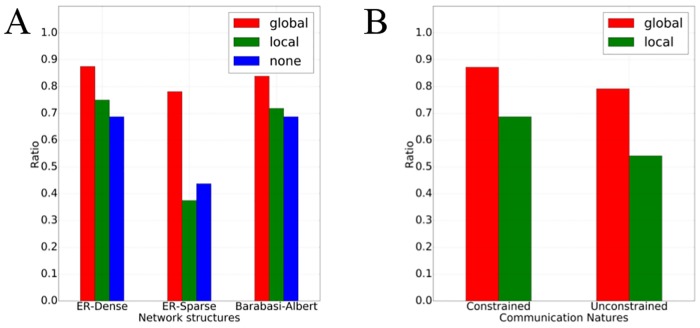
Fraction of games successfully reaching consensus. **A**: differences in success rate of none, local, and global communication grouped by network topology. **B**: differences in success rate for constrained and unconstrained communication, in local and global treatments.

We find that local communication provides virtually no benefit over no communication. Specifically, 60% of all games were solved (subjects reached global consensus) when no communication was allowed, while 61% of games were solved in the local communication treatments. In contrast, when subjects were allowed to communicate across local neighborhood boundaries (global communication), they solved over 83% of the games, a significantly higher fraction than either no or local communication (*p* < 0.005 for both comparisons). Fig 2A shows that this observation is consistent across network topologies: global communication systematically dominates the other forms. The difference is particularly dramatic in the sparse topology, where global communication exhibits nearly double the success rate of local and none treatments. Moreover, these results are also consistent with or without color preference incentives (where people obtain a higher payout for consensus on one color rather than the other).

While communication (when only involving local communities) does not significantly improve performance overall, as compared to no communication at all, there is one measure on which it fairs very poorly compared even to no communication: robustness to changes in network topology. Specifically, we used the ANOVA test to evaluate the significance of variation in performance (fraction of games reaching consensus) across network topologies. We find that both no communication, and global communication, do not exhibit statistically significant variation across networks, although the F-measure for no communication is higher than global (1.39 compared to 0.52). Local communication, however, varies rather dramatically, with F-measure over 6 (*p* < 0.002). In other words, local communication appears to significantly amplify the impact of network structure on coordination.

### Imposing constraints on communication

Our next investigation considers imposing severe constraints on the nature of messages people could send to one another. In particular, previous research, as well as common intuition, would suggest that natural language is a significant mediator of success in human coordination tasks [8–10]. This suggests a hypothesis that constrains should significantly degrade ability of subjects to coordinate. We found that the opposite is true: overall, approximately 67% of games with unconstrained communication were solved, compared with 77% of games solved when only a single type of message could be sent (counts of the two colors in one’s neighborhood) (comparison was significant at *p* < 0.05). Fig 2B shows this to be consistently the case for both local and global communication settings. However, we found that the primary difference arises in BA networks; indeed, this is the only topology on which the difference was significant (*p* = 0.001).

### Individual behavior: Do people respond to messages?

The findings above are extremely surprising, and we now attempt to understand them by analyzing the micro-behavior of individuals in these games. Our overarching question is: do humans behave in fundamentally different ways across these communication settings, or is their behavior consistent, and what differs is the nature of the information conveyed through messages? To address this question, we developed a parametric model of behavior, making use of the following parameters which we hypothesize were the primary observable drivers of individual behavior:

1. **Game stage:** we divided the game into three stages, beginning, middle, and end; the latter two stages (middle, end) were represented as binary variables (the beginning becoming the default).
2. **Number of neighbors (neighbors):** the number of neighbors of a player.
3. **Fraction of neighbors choosing a different color (opposite color):** the fraction of a player’s neighbors who are choosing a different color from the decision maker.
4. **Relative excess of received messages promoting different color over the same color (opposite message):** the count of messages received that suggest using a different color less the count of messages promoting the same color as currently chosen by the decision maker, measured over the previous 15 seconds.
5. **Preference for currently chosen color (prefer current):** whether the player actually prefers if their currently chosen color becomes the consensus choice.

We then discretized time at 1 second intervals, and used a logistic regression to predict the probability that an individual will change their color in the next 10-second interval. We developed 5 such models, one for no communication, and 4 for the four combinations of communication forms (local vs. global) and existence of communication constraints (unconstrained vs. constrained), with all variables normalized to facilitate cross-variable and cross-model comparison.

The results, presented in Table 1, suggest that the behavior is broadly consistent across the different settings. Having a greater fraction of neighbors with and receiving more messages advertising the opposite color increases, while the player prefering their current color reduces the chances that the player will change their color, in all communication settings. An intriguing observation is that the prevalence of messages advertising the color not currently chosen have the greatest impact on an individual’s decision to switch, in most cases far greater than any other factor. Indeed, most surprisingly, it appears to be the strongest factor in local communication, even though we have found it to offer little improvement in facilitating coordination. Similarly, the impact of such messages on decisions only seems to diminish as we introduce constraints. What this strongly suggests is that it is the information content of messages, rather than behavior in response to these, that explains our aggregate observations.

**Table 1:** Coefficients of a logistic regression separated by communication form/structure treatments.

	None	Local	Global	Local (constrained)	Global (constrained)
Intercept	−1.61***	−27.71***	−17.35***	−10.85***	−6.90***
Mid-game	0.05	0.27***	−0.09*	0.05	0.25***
End-game	−0.51***	−0.49***	−0.91***	−0.53***	−0.88***
Neighbors	−2.05***	−1.28***	0.26*	−0.63***	0.32*
Opposite Color	2.20***	2.43***	2.23***	2.54***	2.45***
Opposite Message	NA	43.30***	24.70***	14.83***	7.06***
Prefer Current Color	−0.33***	−0.34***	−0.02	−0.56***	−0.26***

Overall, coefficients are qualitatively consistent across treatments, suggesting that it is the information conveyed in messages that is largely responsible for our aggregate findings.

* *p* < 0.1

** *p* < 0.01

*** *p* < 0.001.

Analysis of individual behavior offers one more noteworthy insight: the importance of an individual’s color preferences diminishes from none and local communication, to global. This suggests that the ability to exchange messages outside of one’s immediate community appears to reduce selfish behavior in global coordination tasks, in favor of increased salience of common interest. This may be another, secondary, factor that helps explain the superior performance on the networked coordination task under global communication.

### Information content in communication

To explore our hypothesis that information content largely explains the relative ineffectiveness of local communication, and effectiveness of global, we consider the extent to which messages received convey important information about global state. To begin, we found that the correlation between message skew in favor of opposing color and global prevalence of that color is much higher in global than local communication (0.68 vs. 0.32). While much smaller, messages in local communication settings appear to still significantly correlate with global state. To explore this issue in greater depth, we developed a quantitative measure of *marginal information* about global state conveyed by messages over time. At the high level, this measure computes how much closer to global state a recipient’s observed information is after receiving messages over a fixed unit of time than they were prior to these messages (based on both choices by immediate neighbors, as well as messages received in the past).

More precisely, in order to measure how informative communication is in the local and global communication treatments, we consider marginal information of messages over a fixed interval Δ. We wish for this measure to capture the following intuition: the informational value of messages received during this interval should be about how much closer it brings the information state of a node to global state, relative to information the node already possesses. We therefore use the following measure which captures this intuition. For a node *i*, define local state at time *t* as follows:

$$\begin{array}{c} r_{t}^{i}=\frac{R_{t}^{i}+R_{M_{t}}^{i}}{R_{t}^{i}+R_{M_{t}}^{i}+G_{t}^{i}+G_{M_{t}}^{i}}, \end{array}$$ (1)

where $R_{t}^{i}$ is the number of Red and $G_{t}^{i}$ the number of Green colored neighbors in the immediate neighborhood of *i*, and $R_{M_{t}}^{i}$ and $G_{M_{t}}^{i}$ the numbers of red and green colors reported in messages received over a fixed time period prior to *t*. This is the local state prior to messages received in the time interval [*t*, *t* + Δ]. Now, consider *new* messages *M* received by *i* in this time interval, with $R_{M}^{i}$ and $G_{M}^{i}$ the count of red / green colors reported by *M*. We define this new information as

$$\begin{array}{c} r_{t\cup M}^{i}=\frac{R_{t}^{i}+R_{M_{t}}^{i}+R_{M}^{i}}{R_{t}^{i}+R_{M_{t}}^{i}+R_{M}^{i}+G_{t}^{i}+G_{M_{t}}^{i}+G_{M}^{i}}. \end{array}$$ (2)

Our target is global state, defined with respect to *R* and *G*, overall counts of Red / Green messages in the entire network at time *t* + Δ, as the fraction of red in the network at this time:

$$\begin{array}{c} g_{t+\Delta }=\frac{R}{R+G}. \end{array}$$ (3)

Information is defined as the distance to global state. Thus, information before messages is $d_{t}^{i}=\left|r_{t}^{i}-g_{t+\Delta }\right|$, and information with these is $d_{t\cup M}^{i}=\left|r_{t\cup M}^{i}-g_{t+\Delta }\right|$. We then define marginal information as the amount by which messages *M* bring local state closer to global state:

$$\begin{array}{c} MI=\max\{0,d_{t}^{i}-d_{t\cup M}^{i}\}. \end{array}$$ (4)

**Fig 3:**
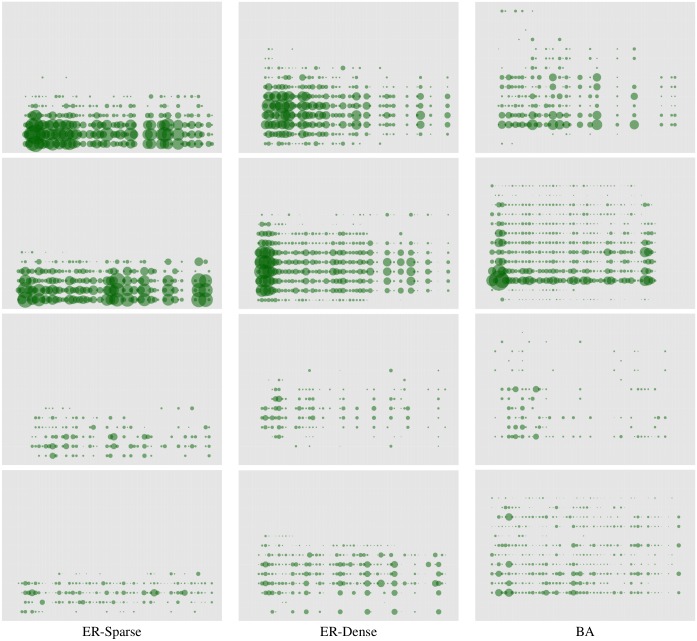
Marginal information conveyed in messages received by nodes over time (x-axis) as a function of their degree (y-axis). **Row 1**: global communication, unconstrained. **Row 2**: global communication, constrained. **Row 3**: local communication, unconstrained. **Row 4**: local communication, constrained. Messages in global communication are considerably more informative. The difference is especially significant in ER-Sparse networks, explaining the rather dramatic advantage of global communication in such settings. Global communication also promotes information equity: lower-degree nodes often obtain considerable information through messages, compensating for lower visibility in the network.

**Fig 4:**
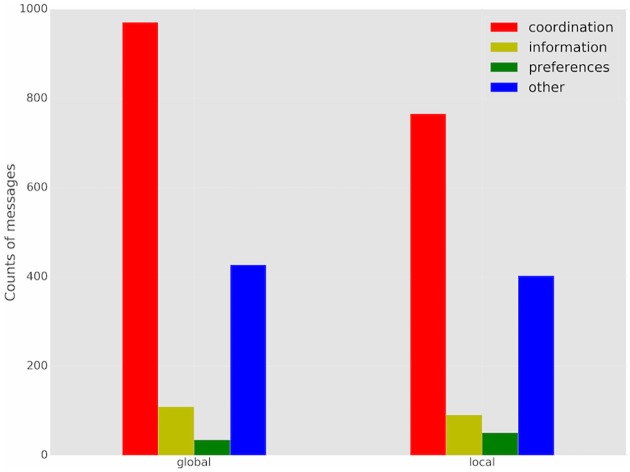
The distribution of counts of messages sent broken down by message category.

Fig 3 demonstrates that marginal information conveyed by messages over time is significantly greater in global communication than local, particularly early in the games. A part of this phenomenon is clearly that more messages are received in global communication treatments. What is considerably more surprising, however, is that significantly more messages are also *sent* in global communication. Fig 4 shows the counts of messages sent, broken down into four *communication categories:* *coordination* category, where a color name is communicated, presumably in an attempt to coordinate on it; *information* category, in which messages simply communicate the number of neighbors choosing each color, as in the constrained communication treatments; *preferences* category, in which player’s communicate their preferences; and *other* category which includes all other messages. By far the most common messages (aside from “other”) were coordination messages naming specific colors. The intent of these appears to have been a directive to others to play the specified color. Over 100 more messages from this category were sent in the global than local communication games. This finding may be connected to our previous observation that common interest is a stronger factor in global communication settings, and players take additional effort to achieve global coordination. Thus, in local communication settings, even though messages have substantial impact on behavior, they bear little additional information as compared to local color visibility, and fewer are sent. Consequently, with global communication, even though relative impact of messages on behavior was somewhat smaller, the overall impact of messages on behavior was substantially more than in local communication settings.

Fig 4 also helps explain the difference observed between constrained and unconstrained communication treatments. Note that messages conveying information were actually relatively infrequent (fewer than 10% of all messages sent). This partially explains why constrained communication settings achieved somewhat higher consensus rates. Indeed, as shown in Fig 3, we can also observe that constrained communication games involved messages which were more informative *earlier* during the game for ER-Dense and BA networks, speeding up consensus. This partially accounts for our observation that the advantage of constrained over unconstrained communication is most significant for BA networks (it is slight, but not significant, in ER-Dense settings).

### Individual communication behavior

To obtain a deeper understanding of individual communication behavior, we now investigate the individual propensities of sending messages from each of the four categories described above: *coordination*, *information*, *preferences*, and *other*. Similar to our analysis of color-change behavior above, we developed a parametric model of communication behavior. This model uses the already defined parameters **mid-game**, **end-game**, **neighbors**, **opposite color**, **opposite message**, and **prefer current color**, as well as 8 additional parameters, corresponding to the number of messages sent and received in the previous 15 seconds for each of the four types of messages. Again, we discretized time at 1 second intervals, but this time we used a multinomial logistic regression to predict the probability that an individual will send a message of a particular category in the next 10 seconds, with *no message sent* being the reference category. If more than one message is sent in the next 10 seconds, we use the category of the earliest message. All variables were normalized to facilitate cross-variable comparison.

The results of the individual communication model are presented in Table 2, and provide several interesting insights. First, there is clear evidence of inertia and/or individual predilection for specific message types: having previously sent coordination or information messages strongly indicates that such messages will be chosen in the future. Interestingly, however, messages about preferences are an exception: it appears that these are restricted to contextual use. Second, there is also a significant evidence of message mimicry: receiving messages from a given category significantly increases the chances of sending a message from the same category. This tendency to imitate messages could potentially be leveraged to improve the ability to coordinate even in unconstrained settings, for example, by inducing specific nodes on the network to send more informative messages, aiming to spark an information cascade. Third, individuals who have sent preference messages are significantly less likely to offer information to their neighbors, opting for coordination messages instead. These individuals appear to be trying to achieve their preferred outcome by persuading their neighbors to choose their preferred color. A related phenomenon can be seen in the significance of “opposite color” (more neighbors choosing a color different from the node’s current choice): in this context, information messages are unlikely, and the node is instead more likely to either explain why their choice is different from the neighbors’ by indicating their preference, or tries to persuade neighbors to switch to their color choice by sending coordination messages.

**Table 2:** Coefficients of a multinomial logistic regression separated by sent message category class.

	coordination	information	preferences	other
Intercept	−2.74***	−6.06***	−8.64***	−6.05***
Mid-game	−0.06***	−0.31***	−0.36***	0.26***
End-game	−0.80***	−1.01***	−1.09***	−0.39***
Neighbors	0.22***	0.38***	0.56***	0.11
Opposite Color	0.24***	−0.34***	0.95***	−0.13**
Opposite Message	1.67***	6.08***	6.84***	5.36***
Prefer Current Color	0.05***	0.20***	0.37***	0.02
Received Coordination Messages	0.79***	−0.53***	0.27	0.47***
Received Information Messages	0.03	17.39***	−19.87***	−13.06***
Received Preferences Messages	1.49***	0.03	5.21***	2.32***
Received Other Messages	−0.38***	−2.00***	−3.98***	1.24***
Sent Coordination Messages	7.85***	0.40	2.33**	6.04***
Sent Information Messages	2.42***	18.25***	4.24*	−0.51
Sent Preferences Messages	0.82**	−6.55***	5.72	−2.29***
Sent Other Messages	0.99***	−1.07**	1.47*	6.20***

The reference class is ‘no message sent’. Only games with unconstrained communication are considered.

* *p* < 0.1

** *p* < 0.01

*** *p* < 0.001.

### Mixed communication treatments

The findings and analysis above suggests that global communication promotes coordination in large part because messages, in aggregate, convey significantly more information. Somewhat surprisingly, information overload does not appear to be an issue at the scale of our experiments. However, many other challenges exist in supporting global communication in coordination tasks, including costs. Moreover, it is likely that with significantly larger systems, information overload would indeed become a major concern. A natural question is, therefore: can we obtain the same informational benefit in supporting coordination through only a subset of globally communicating nodes?

To address this question, we ran a series of additional experiments (totalling 550 games with 156 unique participants) in which we varied the number of globally coordinating individuals (with the rest communicating locally), considering 2, 4, and 10 (in addition to local communication, which corresponds to 0, and global communication treatments above, corresponding to 20). In all cases, all players, including the global communicators, were evenly divided among the two color preferences (when these were relevant). The distribution of other experimental parameters was kept as above.

**Fig 5:**
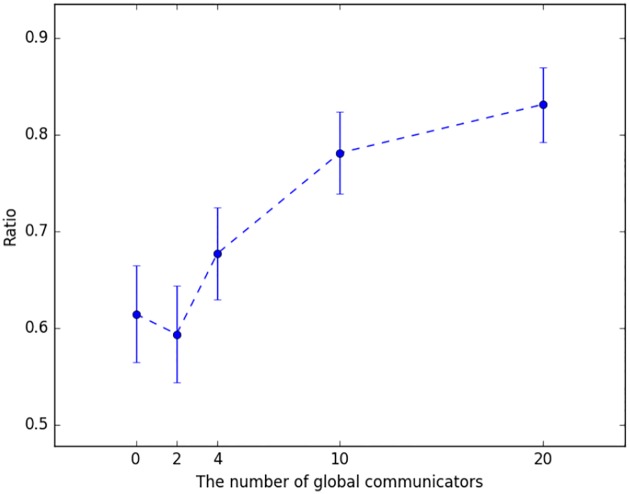
Fraction of games solved (y-axis) as a function of the number of global communicators (x-axis); all other nodes communicate locally.

Given the significance of global information in reaching consensus explored above, one would expect that relatively few global communicators would be sufficient to serve as global information aggregators and disseminators. Fig 5, however, tells a different story. The figure shows the fraction of games solved for 0, 2, 4, 10, and 20 global communicators (the rest of the players being able to communicate only locally). Surprisingly, increasing the number of global communicators from 0 to 2 has virtually no impact (indeed, the success rate drops somewhat, although the drop is not statistically significant). Increasing this number to 4 improves performance only slightly, with the improvement not reaching statistical significance. Only with 10 (50%) global communicators do we see a significant increase in performance, although it still lags somewhat behind fully global communication settings.

### Communication advantage and equity

As we contemplate decentralized coordination with only a subset of globally communicating individuals, an important consideration that arises when preferences for consensus color differ is equity: will global communicators use their power to steer consensus towards their preference, against that of the majority. Indeed, this consideration is significant in public policy as well: communication ability is extremely asymmetric, with some individuals having a far broader forum than the overwhelming majority of others, and the resulting ability to have public opinion converge to align with their interests, and potentially against those of the majority, is a major concern.

To explore this issue, we consider how much of a role network topology plays in either facilitating, or inhibiting, the power of a small globally communicating minority to influence outcomes. We hypothesized, in particular, that a highly cohesive globally communicating minority would have significant power, but would be somewhat weaker when the network has a high degree of clustering as compared to networks in which non-minority nodes form an Erdos-Renyi-like topology. To explore this, we follow the idea introduced by Judd et al. [22], where a network is initially a set of 4 loosely connected cliques of 5 nodes each (specifically, the network is a line of 4 cliques, the two interior cliques are connected by one edge to both their immediate neighbors, whereas the two outer cliques are connected only to the left/right neighbor). We then introduce a parameter *q* ∈ [0, 1], such that each edge between two non-global-communicators is rewired with probability *q* to a randomly selected node on the network (in addition, all edges connecting the cliques remain intact to ensure that the graph always remains connected). Thus, when *q* is small, the network remains highly clustered, whereas a large *q* leads to nearly Erdos-Renyi networks, with the exception of the global communicators, who retain their internal clique structure. Nodes which do not communicate globally now have two possibilities: they may be able to communicate locally (that is, only their immediate neighbors can receive their messages), or not at all. We refer to the former possibility as *GL* (global-local), and the latter as *GN* (global-none). These two possibilities induced a 6x2 design: we varied *q* ∈ {0, 0.1, 0.2, 0.4, 0.6, 1}, as in [22], and varied communication ability of the majority to be local, or inhibited altogether. Altogether, we ran 375 games involving 158 unique participants. Throughout, the preferred color of the globally communicating minority was *Red*, while the majority preference was *Green*. Below we define *R* to be the number of players choosing red at the end of the game and *G* the number of players choosing green. Consequently *P* = *R* − *G* (*P* for *power*) quantifies the number of players choosing the minority preference, which we take to indicate the ability of the globally communicating minority to influence overall choices. Note that *P* > 0 implies that the minority is able to sway a large proportion (at least 1/3) of the majority away from their preferred color choice, to support the preference of influential minority.

Our two hypotheses were: 1) globally communicating minority would have more power for high values of *q* than low, and 2) globally communicating minority would have more power when others do not communicate, than when others communicate locally. The results of our experiments support the second hypothesis, but not the first. Specifically, minority power, *P*, was 7.0 for high-*q* settings (*q* ∈ {0.4, 0.6, 1}) and 4.2 for low-*q* settings (*q* ∈ {0, 0.1, 0.2}). While there is a difference between the two settings, it is not statistically significant. Looking at the differences between majority with local vs. no communication, however, *P* was 1.9 for the former, and 9.4 for the latter, for a highly significant difference (*p* < 0.001). This impact of the ability to communicate locally is particularly striking in the light of our results above: while local communication appears to play little role in facilitating consensus, it plays a major role in facilitating *equity* in outcomes.

**Fig 6:**
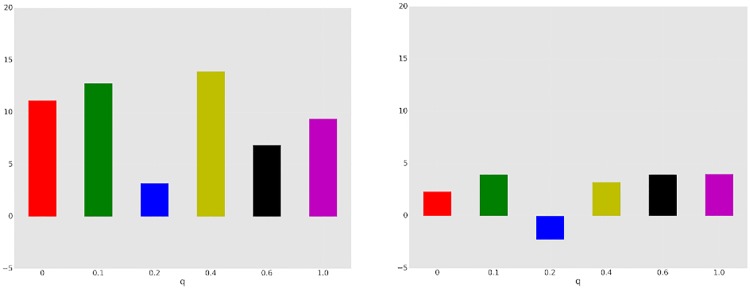
*P* for *GN* treatments (left) and *GL* treatments (right).

**Fig 7:**
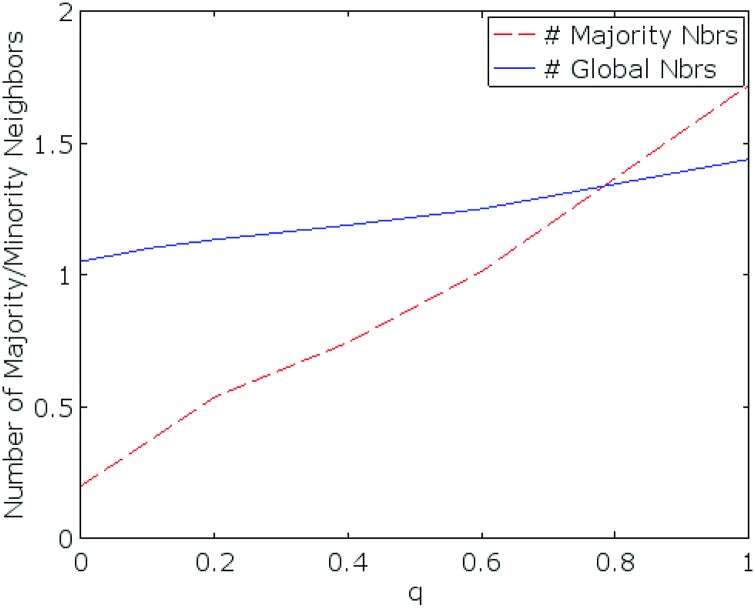
The average number of neighbors of “majority” nodes who are global communicators (Red, dashed), and the average number of neighbors of global communicators who are “majority” nodes (Blue, solid), as a function of *q*.

To appreciate why the high vs. low *q* distinction is not clearly borne out, we visualize *P* as a function of network topology *q* for *GN* and *GL* settings in Fig 6. We can see that the minority power *P* for *GN* treatments dominates *P* for *GL* games over all topologies (values of *q*), typically by a substantial margin. However, looking across all values of *q*, there is no unambiguous trend, even though there is some difference as we aggregate across the three smallest and three largest values of *q*. The most provocative is the fact that *q* = 0.2 appears to be distinct from the other network topologies: in all other cases, global communicators are consistently able to sway many of the other nodes towards their color preference in at least the *GN* treatment, and typically both in *GN* and *GL*. This observation is particularly surprising because there is no single property of the network topology which easily explains it. For example, average diameter monotonically decreases with *q*, as does clustering coefficient. To make sense of the results, however, we note that there are two quantities that both increase monotonically with *q*, but likely have the opposite effect: the average number of neighbors of “majority” nodes who are global communicators, and the average number of neighbors of global communicators who are “majority” nodes (see Fig 7). The effect of the first is that global communicators have greater *direct* influence on others (through observed color choices). The effect of the second, however, is that majority nodes have increasing influence on global communicators. Note that this is not just direct influence: in local communication treatments, global communicators are exposed to more messages sent by majority nodes. It appears that for *q* = 0.2 these opposing topological effects are less favorable to global communicators.

**Fig 8:**
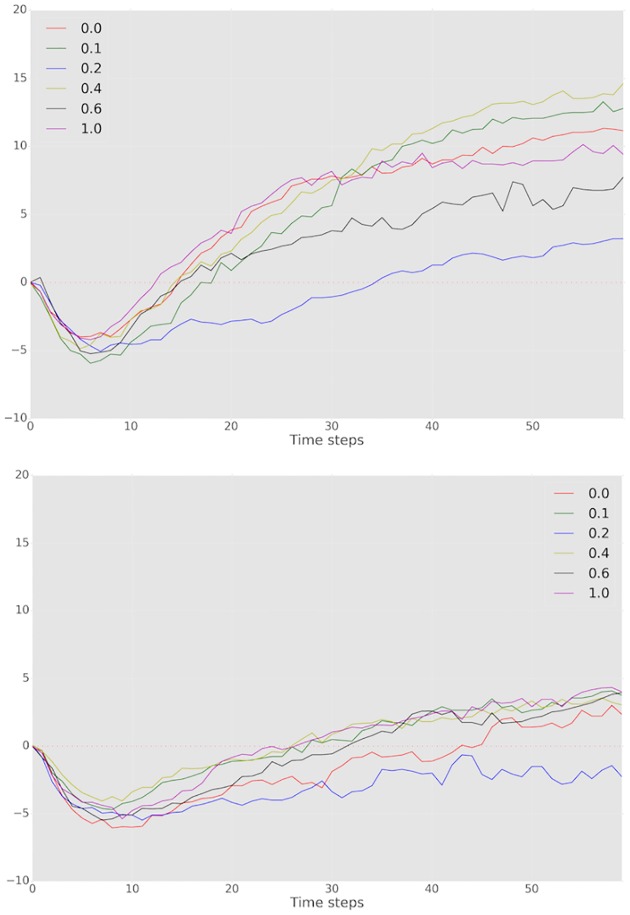
*P* over time for *q* and *GN* (top) and *GL* (bottom) treatments.

Fig 8 allows us to look at the evolution of minority power *P* as the games progress. The initial *P* = 0 simply reflects that no one has yet chosen a color. As initial color choices are made, they reflect the overall balance of preference, resulting in *P* < 0. Remarkably, the ability to communicate globally reverses this trend towards majority preference, so that by mid-game *P* > 0 in most cases. It is noteworthy that *q* = 0.2 is the one case in which the trend is never fully reversed. However, in *GN* experiments, it appears that eventually consensus would indeed emerge at minority preference even for *q* = 0.2, whereas local communication treatments exhibit a stable trend where *P* is not trending up in the long term for *q* = 0.2.

**Fig 9:**
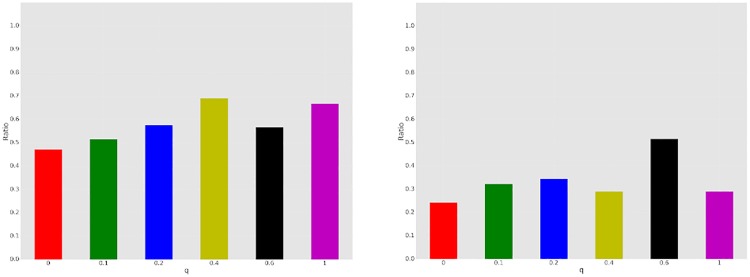
Proportion of games solved for *GN* treatments (left) and *GL* treatments (right).

Finally, we return to our original question: how is the proportion of instances solved affected by problem parameters, which in this case involve network topology (*q*) and the mode of communication for the majority (local or none). Fig 9 offers a sobering picture: significantly more instances are solved in *GN* than *GL* settings; communication actually inhibits consensus (the difference is significant, with *p* < 0.001)! In the context of our observations above, this actually makes sense: allowing majority to communicate locally increases equity, but it also increases the conflict between the two opposing preferences as a result, making consensus more difficult to reach.

## Conclusion

Much prior literature demonstrates, often unambiguously, the substantial value that communication has in facilitating coordination. This seems almost a foregone conclusion when one considers the importance of communication in one’s everyday small-scale coordination activities, ranging from who picks up the kids from school to how a particular complex task should be split among several workers. Game theoretic literature has explored extensively the *strategic* role of “cheap-talk” communication, taking for granted the role it serves in providing valuable information about the state of the world. Our experiments explored communication as embedded in a coordination task, allowing subjects to make decisions and communicate in real time, and we systematically investigated the impact that different constraints on communication play in its value to the coordination task. We found that from a behavioral standpoint, people indeed “respond” to messages that they receive: specifically, they are significantly more likely to change their decision if it conflicts with received messages. This behavioral trait is consistent across all communication treatments. The key differentiator is how *informative* communication is: when people can also discuss the task locally, little information about global state is ultimately conveyed—too little to improve coordination performance. In contrast, global communication is clearly far more informative, and that ultimately leads to improved performance. This consideration of information conveyed in communication has not figured significantly in prior literature, even though realistic communication contexts are, typically, local. Consequently, our findings suggest that communication through formally constructed local channels may be insufficiently effective in promoting global coordination, and entities, such as media and government, with the ability to reach a broad array of the population have a critical role to play in facilitating coordination. Moreover, we find that a globally communicating minority with preferences opposed to the rest can consistently steer outcomes towards their preference (and counter preferences of the majority). However, the ability to communicate locally does significantly mitigate the resulting inequity, albeit at the expense of increased conflict and reduced success rate in reaching global consensus.

While our work is in the context of a global coordination task, the implications may have broader consequences. For example, one could view diffusion of competing technologies which are economic substitutes through a similar lens of global coordination in a population, particularly when there are significant network externalities. Our findings suggest that local communication may not be sufficient to reduce the inefficiency due to miscoordination. However, this parallel is only limited: diffusion of substitute technologies is economically more nuanced than pure coordination, as non-zero utility is achieved even when coordination fails.
